# Supplementary figures and images for: Extra high superoxide dismutase in host tissue is associated with improving bleaching resistance in “thermal adapted” and Durusdinium trenchii-associating coral
Source: PeerJ. 2022 Jan 12;10:e12746. doi: 10.7717/peerj.12746 (PMC8760857; doi:10.7717/peerj.12746)

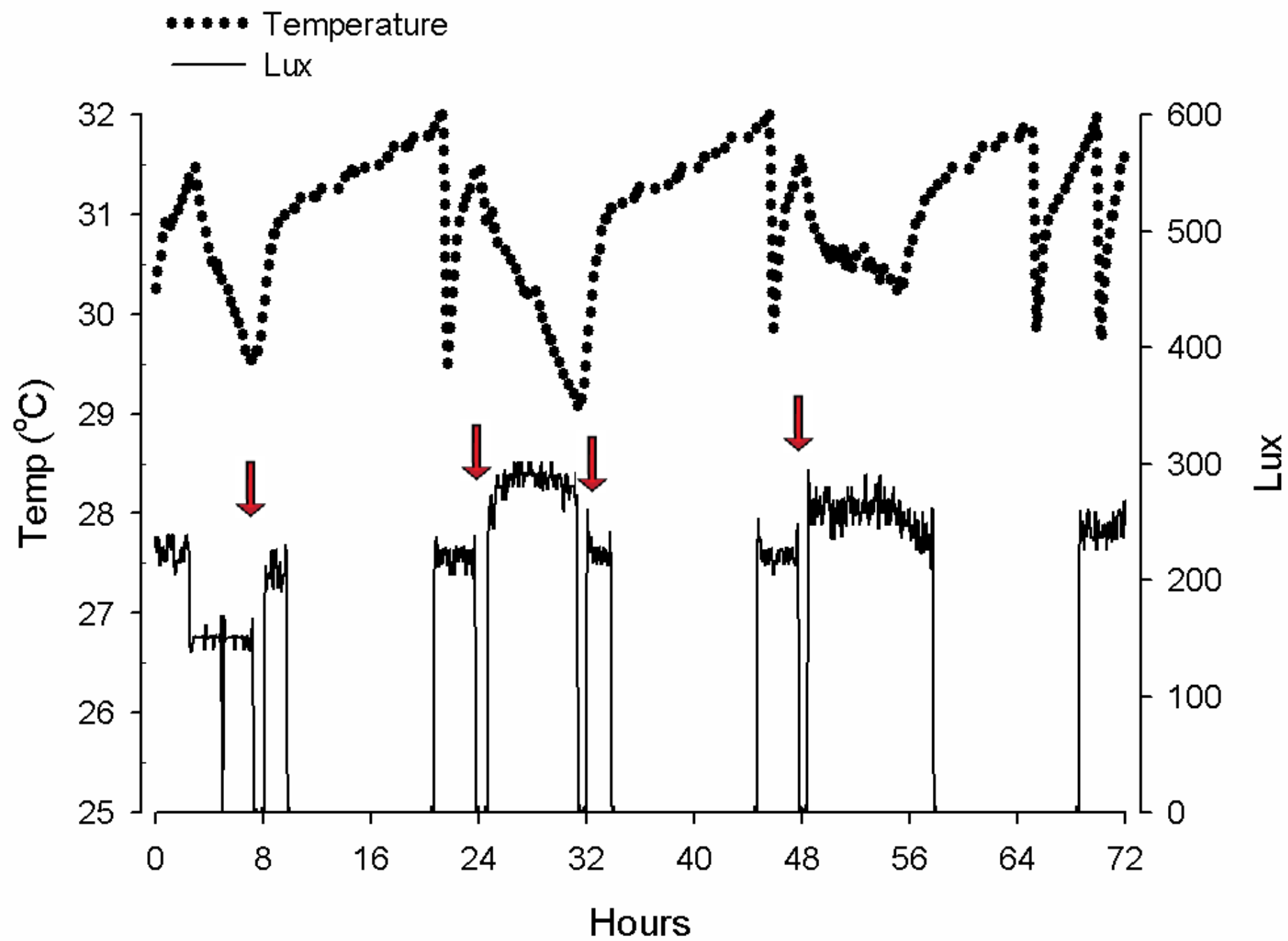

Supplement: Supplemental Information 1 — Light was turned off during the illumination regime, as indicated by the red arrow, to conduct dark adaption before PAM quantum yield measurements. [file peerj-10-12746-s001.pdf]

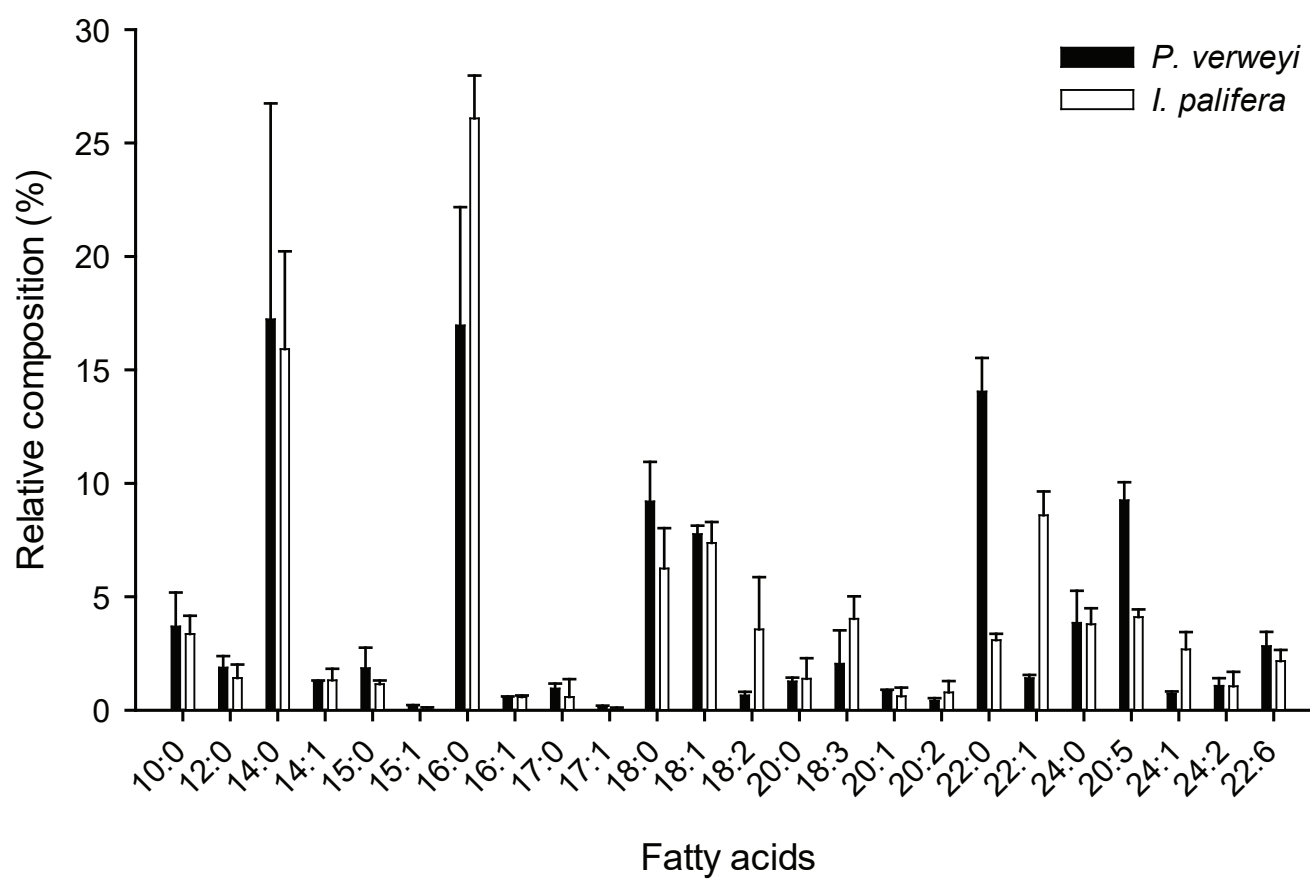

Supplement: Supplemental Information 2 [file peerj-10-12746-s002.pdf]

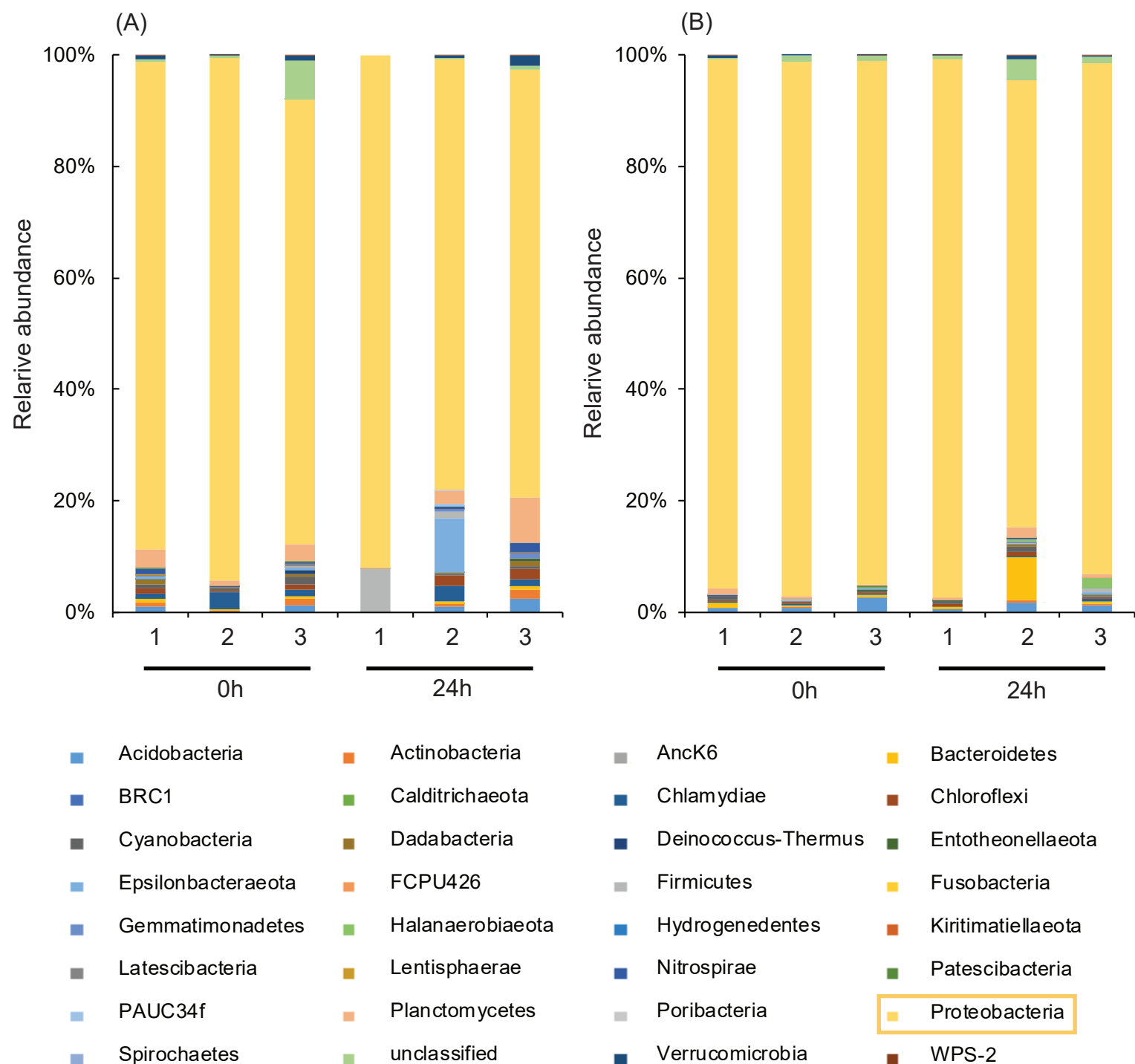

Supplement: Supplemental Information 3 — Numbers (1, 2, 3) represent three colony replicates. (A) contains the samples from Isopora palifera and (B) from Platygyra verweyi. In the legend, the most dominant phylum is marked in a box. [file peerj-10-12746-s003.pdf]

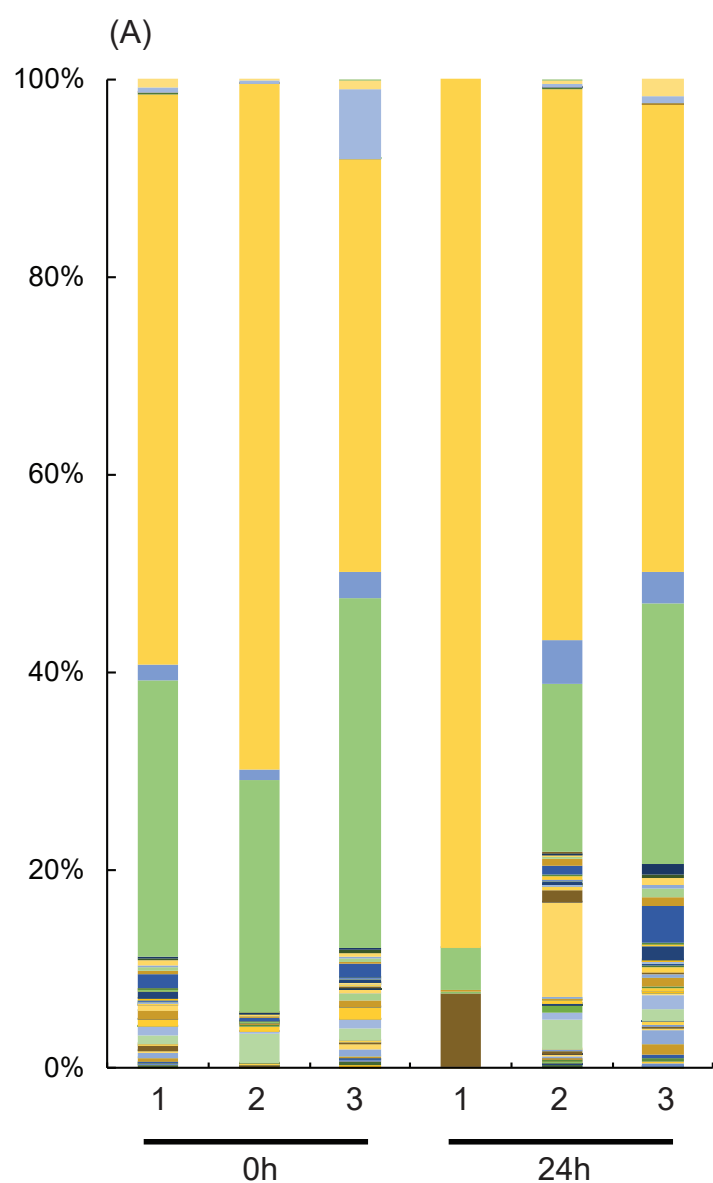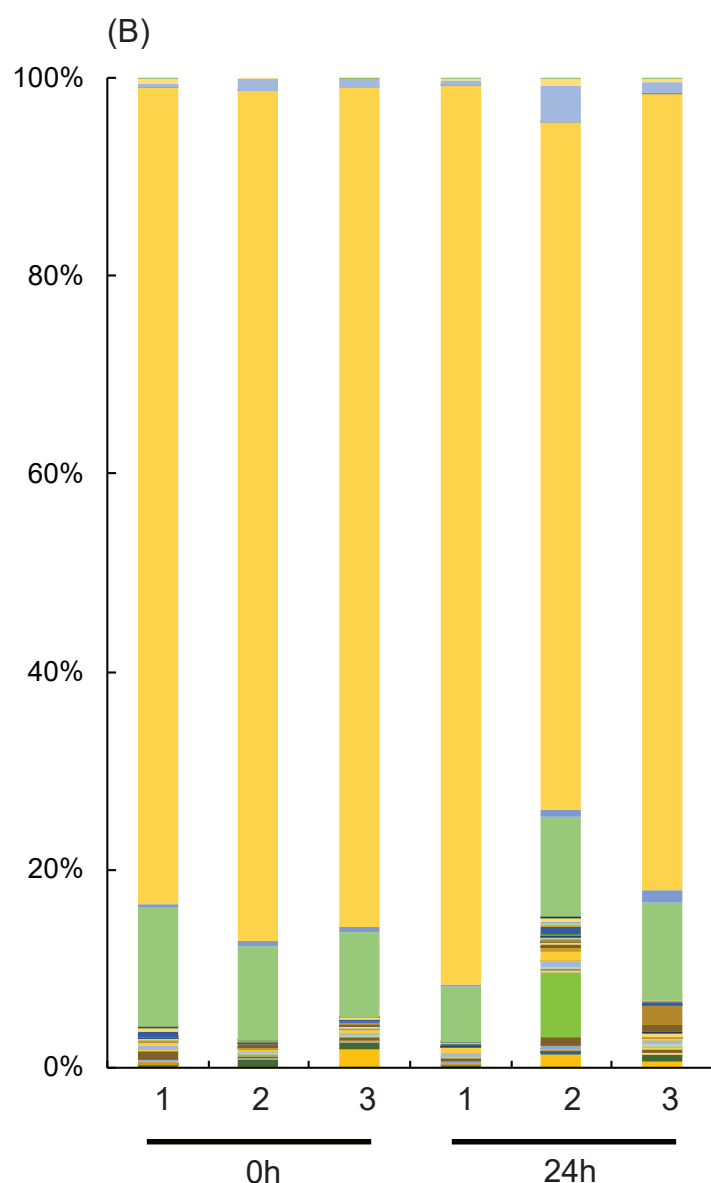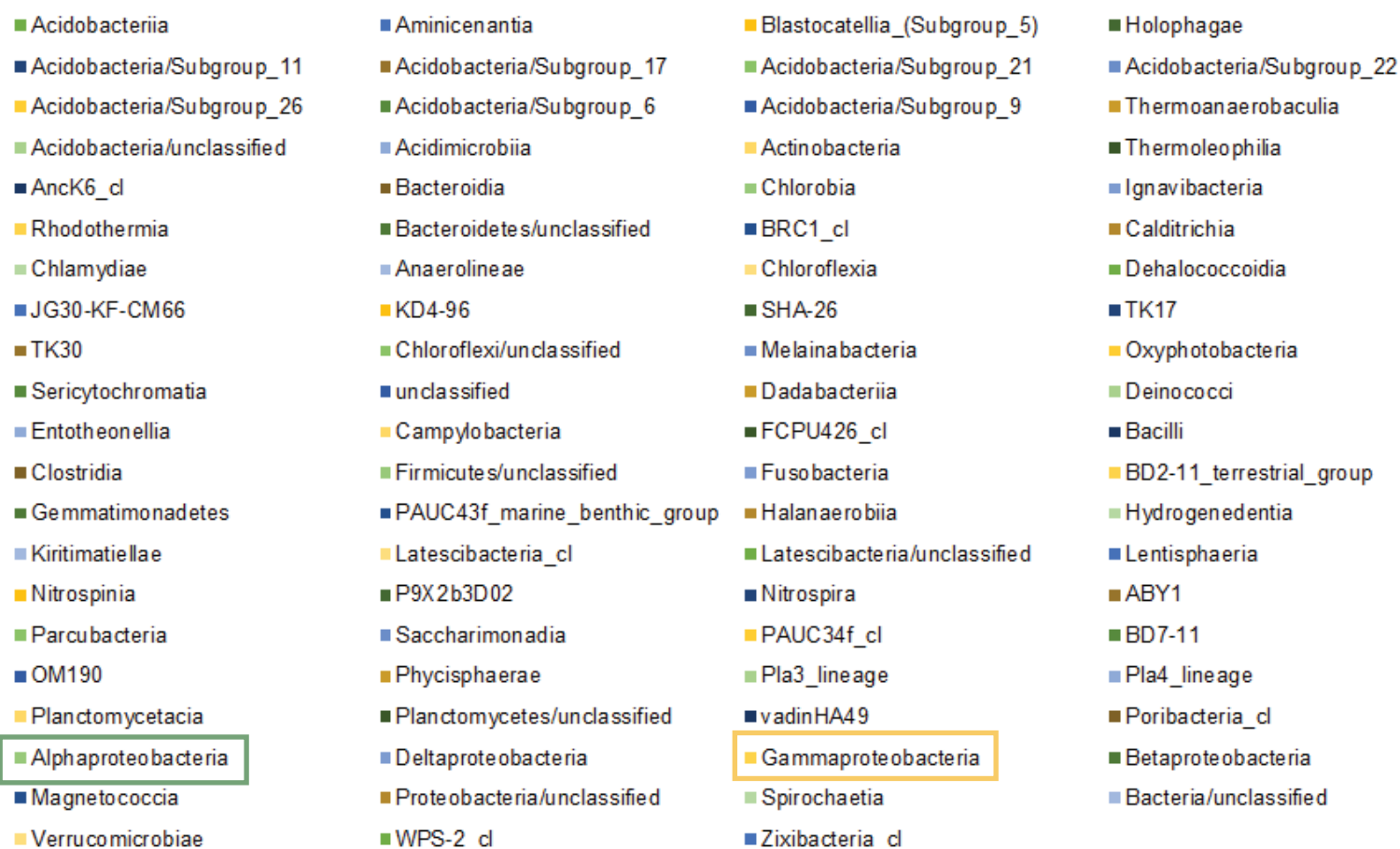

Supplement: Supplemental Information 4 — Numbers (1, 2, 3) represent three colony replicates incubated at 31 °C for 0 and 24 h. (A) contains samples from Isopora palifera and (B) from Platygyra verweyi. In the legend, the two most dominant classes are marked in a box. [file peerj-10-12746-s004.pdf]
