# Supplementary material for: Extra high superoxide dismutase in host tissue is associated with improving bleaching resistance in “thermal adapted” and Durusdinium trenchii-associating coral
Source: PeerJ. 2022 Jan 12;10:e12746. doi: 10.7717/peerj.12746 (PMC8760857; doi:10.7717/peerj.12746)
Supplement: Supplemental Information 5 — Samples from 3 replicate colonies (1, 2, and 3) were collected before treating with antibiotics or Endozoicomonas infection (Pre-Exp) and 0, 24, 48, and 72 h heating at 31 °C for analyses. “Endo” represents the detection of Endozoicomonas. [file peerj-10-12746-s005.pdf]

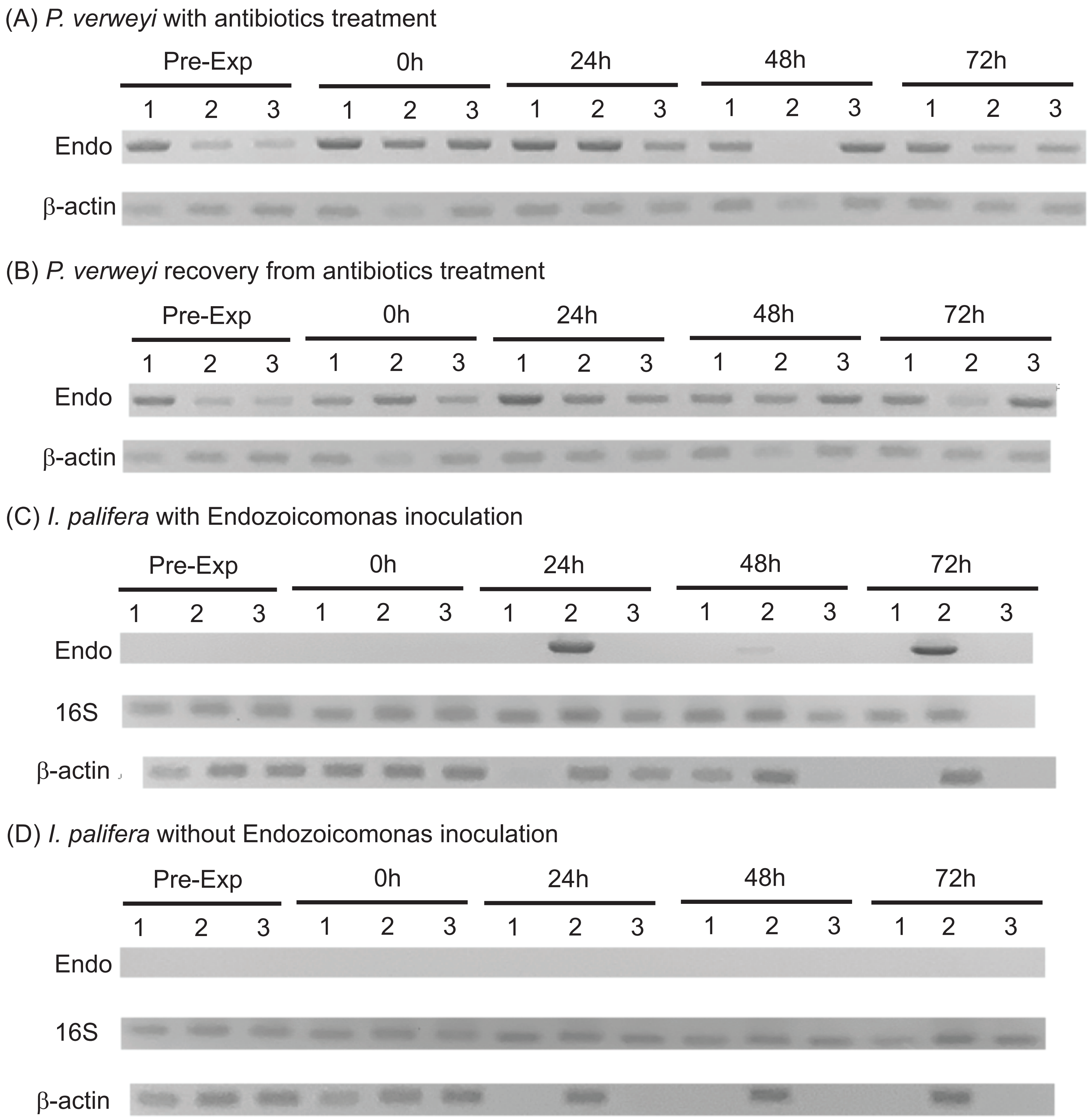
(A) P. verweyi with antibiotics treatment
Pre-Exp
0h
24h
48h
72h
1 2 3 1 2 3 1 2 3 1 2 3 1 2 3
Endo
β-actin
(B) P. verweyi recovery from antibiotics treatment
Pre-Exp
0h
24h
48h
72h
1 2 3 1 2 3 1 2 3 1 2 3 1 2 3
Endo
β-actin
(C) I. palifera with Endozoicomonas inoculation
Pre-Exp
0h
24h
48h
72h
1 2 3 1 2 3 1 2 3 1 2 3 1 2 3
Endo
16S
β-actin
(D) I. palifera without Endozoicomonas inoculation
Pre-Exp
0h
24h
48h
72h
1 2 3 1 2 3 1 2 3 1 2 3 1 2 3
Endo
16S
β-actin
