# Supplementary material for: Extra high superoxide dismutase in host tissue is associated with improving bleaching resistance in “thermal adapted” and Durusdinium trenchii-associating coral
Source: PeerJ. 2022 Jan 12;10:e12746. doi: 10.7717/peerj.12746 (PMC8760857; doi:10.7717/peerj.12746)
Supplement: Supplemental Information 6 — The data are mean ± SD from three colony replicates. [file peerj-10-12746-s006.docx]

Table S1. Fatty acid (FA) composition of menthol-bleached *Platygyra verweyi* and *Isopora palifera*. The data are mean ± SD from three colony replicates.

|  | saturated | mono-unsaturated | poly-unsaturated |
| --- | --- | --- | --- |
|  | % | | |
| *P. verweyi* | 70.9 ± 2.8 | 12.9 ± 0.4 | 16.2 ± 2.5 |
| *I. palifera* | 63.0 ± 2.0 | 21.3 ± 2.6 | 15.7 ± 1.6 |
|  | *t*_4_ = 3.921 P<0.05 | *t*_4_ = -5.486  P<0.01 | *t*_4_ = 0.304  P>0.05 |
