# Supplementary material for: Extra high superoxide dismutase in host tissue is associated with improving bleaching resistance in “thermal adapted” and Durusdinium trenchii-associating coral
Source: PeerJ. 2022 Jan 12;10:e12746. doi: 10.7717/peerj.12746 (PMC8760857; doi:10.7717/peerj.12746)
Supplement: Supplemental Information 7 — Treatments are as described in Table 1. Pre-treatment SOD activity (U mg−1) of P. verweyi and I. palifera were 84 ± 28 and 12 ± 2, respectively, and CAT activity (µmol min−1mg−1) of P. verweyi and I. palifera were 1,493 ± 617 and 1,230 ± 309, respectively. Data are expressed as relative percentages of enzyme activity in each treatment to that in pre-treated colonies. [file peerj-10-12746-s007.docx]

Table S2. Changes in the activity of superoxide dismutase (SOD) and catalase (CAT) in *Platygyra verweyi* and *Isopora palifera* with or without endogenous *Endozoicomonas* sp. or infected *E. montiporae*. Treatments are as described in Table 1. Pre-treatment SOD activity (U mg^-1^) of *P*. *verweyi* and *I. palifera* were 84 ± 28 and 12 ± 2, respectively, and CAT activity (μmol min^-1^mg^-1^) of *P*. *verweyi* and *I. palifera* were 1,493 ± 617 and 1,230 ± 309, respectively. Data are expressed as relative percentages of enzyme activity in each treatment to that in pre-treated colonies.

| hours at 31°C |  | SOD | | | | |  | CAT | | | | | |
| --- | --- | --- | --- | --- | --- | --- | --- | --- | --- | --- | --- | --- | --- |
|  |  | *P. verweyi* | |  | *I. palifera* | |  | *P. verweyi* | | |  | *I. palifera* | |
|  |  | +Ab | recovery from +Ab |  | + Endo. | - Endo. |  | +Ab | recovery from +Ab | | | + Endo. | - Endo. |
| (%) | | | | | | | | | | | | | |
| 0 |  | 87 ± 15 | 93 ± 11 |  | 121 ± 18 | 93 ± 39 |  | 94 ± 10 | | 112 ± 15 |  | 182 ± 65 | 142 ± 41 |
| 24 |  | 90 ± 15 | 92 ± 26 |  | 117 ± 8 | 77 ± 15 |  | 124 ± 36 | | 116 ± 26 |  | 233 ± 13 | 180 ± 43 |
| 48 |  | 87 ± 21 | 73 ± 14 |  | 102 ± 31 | 84 ± 21 |  | 136 ± 44 | | 113 ± 7 |  | 227 ± 37 | 206 ± 49 |
| 72 |  | 82 ± 19 | 70 ± 30 |  | 236 ± 122 | 73 ± 14 |  | 184 ± 55 | | 97 ± 33 |  | 133 ± 97 | 194 ± 38 |
| 72h at 25°C |  | 113 ± 9 | 81 ± 11 |  | 117 ± 21 | 74 ± 28 |  | 165 ± 9 | | 110 ± 39 |  | 188 ± 30 | 164 ± 31 |
