# Supplementary material for: Extra high superoxide dismutase in host tissue is associated with improving bleaching resistance in “thermal adapted” and Durusdinium trenchii-associating coral
Source: PeerJ. 2022 Jan 12;10:e12746. doi: 10.7717/peerj.12746 (PMC8760857; doi:10.7717/peerj.12746)
Supplement: Supplemental Information 8 [file peerj-10-12746-s008.pdf]

Platygyra-b-actin

TTCCTTGGAATGGAATCTGCAGGTATCCACGAGACCACATATAATTCCATCATGAAGTGCGACG
TGGACATCCGAAAGGACTTGTACGCCAACACAGTGTTGTCTGGTGGCTCCACGTACCCAGGA
ATCGCTGACAGAATGCAGAAAGAAATCACTTCGC

Isopora- b-actin

TTCCTTGGAATGGAATCTGCTGGAATCCACGAGACCACGTATAACTCCATCATGAAGTGCGAT
GTGGATATCCGTAAGGACCTGTACGCCAACACAGTATTATCCGGTGGCTCTACCATGTATCCTG
GAATTGCTGACAGAATGCAGAAAGAAATCACTTCGC
